# Classification of precipitation types in Poland using machine learning and threshold temperature methods

**DOI:** 10.1038/s41598-023-48108-2

**Published:** 2023-11-25

**Authors:** Quoc Bao Pham, Ewa Łupikasza, Małarzewski Łukasz

**Affiliations:** Faculty of Natural Sciences, Institute of Earth Sciences, University of Silesia in Katowice, Będzińska Street 60, 41-200 Sosnowiec, Poland

**Keywords:** Atmospheric dynamics, Projection and prediction

## Abstract

The phase in which precipitation falls—rainfall, snowfall, or sleet—has a considerable impact on hydrology and surface runoff. However, many weather stations only provide information on the total amount of precipitation, at other stations series are short or incomplete. To address this issue, data from 40 meteorological stations in Poland spanning the years 1966–2020 were utilized in this study to classify precipitation. Three methods were used to differentiate between rainfall and snowfall: machine learning (i.e., Random Forest), daily mean threshold air temperature, and daily wet bulb threshold temperature. The key findings of this study are: (i) the Random Forest (RF) method demonstrated the highest accuracy in rainfall/snowfall classification among the used approaches, which spanned from 0.90 to 1.00 across all stations and months; (ii) the classification accuracy provided by the mean wet bulb temperature and daily mean threshold air temperature approaches were quite similar, which spanned from 0.86 to 1.00 across all stations and months; (iii) Values of optimized mean threshold temperature and optimized wet bulb threshold temperature were determined for each of the 40 meteorological stations; (iv) the inclusion of water vapor pressure has a noteworthy impact on the RF classification model, and the removal of mean wet bulb temperature from the input data set leads to an improvement in the classification accuracy of the RF model. Future research should be conducted to explore the variations in the effectiveness of precipitation classification for each station.

## Introduction

The ability to discriminate precipitation phase is important due to the different impacts of snowfall and rainfall on an array of environmental processes^1–3^ and several aspects of the industry, including transportation, agriculture, and farming^4,5^. The frequency and type of precipitation are just as important as their quantity and intensity in understanding the health of the ecosystem^6–8^ and the seasonality of hydrological and energy cycles^1–3,9–15^ which is crucial for decrease uncertainty in hydrological models^16–20^ and has a significant effect on water management^7,8,21^. A particular role belongs to the solid precipitation vital for snow cover development, which indirectly modifies radiation balance (increased albedo) and impacts large-scale climate dynamics^22–24^, stream flow and hydrological drought occurrences in spring and summer, snowmelt flooding^25^ and winter sports and recreation^26^. In Central Europe, seasonal variability in runoff and summer low flows are not only a function of low precipitation and high evapotranspiration, but they are also significantly affected by the previous winter snowpack^27^. Heavy snowfall can also be a serious risk to human health, life and property^28–32^ that can be significantly reduced when accurately forecasted. Winter precipitation forecasting relies heavily on the ability to precisely discriminate between winter precipitation types^33–35^ which is a challenging task due to the significant sensitivity of precipitation types to atmospheric conditions^33^. Therefore, evaluation of the role of particular atmospheric parameters (e.g. air temperature, humidity etc.) for forming particular precipitation phases is also useful for precipitation type forecasting. However, such a purpose requires high quality meteorological data for vertical profiles of the lower atmosphere which are available for a very sparse set of stations^36–38^.

Other precipitation phase discrimination attempts were based on single or several atmospheric parameters at the station level, using values averaged over the course of the day^39–42^. Daily data on precipitation phases recognized based on meteorological parameters are useful for climatological and environmental studies due to the lack of visual observations of precipitation phases at many stations. In South Korea out of 780 stations, 22 stations perform the manual observation of precipitation types^43,44^. Data on the precipitation phase, if available, usually covers shorter time periods compared to other meteorological parameters^41^—e.g. from 1979 in China, and from 1966 in Poland. In Poland, at most stations, the available time series of most meteorological elements began in 1951. The longest series of regular meteorological observations started at the end of the 18th or beginning of the 19th century. However such a long series exist for only a few stations and are not included in open databases. Moreover, traditional visual identification of precipitation types is massively replaced by automatic weather stations, which admittedly are able to recognize precipitation phases however with different accuracy and are differently coded, which can produce inhomogeneities in the chronological series^45^.

Novel approaches and procedures are still being developed to precisely identify precipitation phases based on novel data sources like remote sensing^37,42,46,47^ and new statistical tools like machine learning (ML)^34,47,48^. ML is an ideal solution for simulating the intricate interactions between many parameters and the precipitation phase because it does not require any distributional or modeling assumptions to handle multidimensional and complicated nonlinear relationships. However, ML is not without its limitations. There are a lot of distinct algorithms, thereby necessitating meticulous literature reviews and practical experience to align each model effectively with specific tasks. Moreover, the intricate nature of ML models introduces the requirement for parameter tuning, a meticulous process that consumes a substantial amount of time and, if not executed judiciously, can result in overfitting. Furthermore, the challenge of feature selection is also a significant consideration when deploying ML algorithms. Altogether, these procedural intricacies demand a substantial investment of time and the application of expert knowledge. Still, ML was extensively employed in the atmospheric sciences^49–54^. A multinomial logistic regression (MLR) model that used the NWP-driven variables beat the optimized Matsuo scheme and exhibited a 15% gain in accuracy relative to the NWP forecast^44,55^ tested the six following ML models—k-nearest neighbors, logistic regression, support vector machine (SVM), decision tree (DT), random forest (RF), and multi-layer perceptron and using thermodynamic and polarimetric variables, found that RF outperformed the operational technique and displayed the top score. When applying a ML model, optimizing and searching for an appropriate ML model is vital in order to assure both accuracy and computational efficiency in real-time operation^55^.

Accurate identification of the precipitation phase is still an important problem in atmospheric research, particularly climate change studies, and weather prediction that are both vital for society. The goal of this study is to use ML to discriminate snowfall from rainfall based on the lowest number of atmospheric parameters possible. Additionally, this study seeks to (i) identify the thresholds of daily mean air temperatures (*Ta*) and mean wet bulb temperatures (*Tw*) to differentiate between precipitation phases using both traditional and ML methods based on daily precipitation data collected from 40 best quality synoptic stations located across Poland between 1966 and 2020; (ii) explore the relationships between precipitation phases and various meteorological variables and select the optimal set of variables for accurate identification of precipitation phase based on ML; (iii) compare the accuracy of the threshold temperature and the Single Input Random Forest (SIRF) methods in determining precipitation phase to select the method that performs the best.

Although based on Polish data, this study delivers a universal method with respect to region, for identification of precipitation phases and contributes to the knowledge of the factors triggering the occurrence of precipitation phases. The results of this study can be useful for climate change environmental studies.

## Dataset and methodology

### Study area and dataset

Poland is located in Central Europe where the climate is moderate and transitional between oceanic and continental. Poland features varied landscapes, including the mountain ranges in the south with the highest peak reaching 1991 m asl, and lowlands and plains in the central regions, and a coastline along the Baltic Sea to the north. Average annual temperature varies between 7 and 8.5 °C and drops below 5 °C in mountain regions. In winter the warmest western and west-eastern edges of Poland experience air temperatures slightly above 0 °C while in the north-eastern part of the country air temperature drops to below – 3 °C. The record minimum daily air temperature was as low as − 41.0 °C.

**Figure 1:**
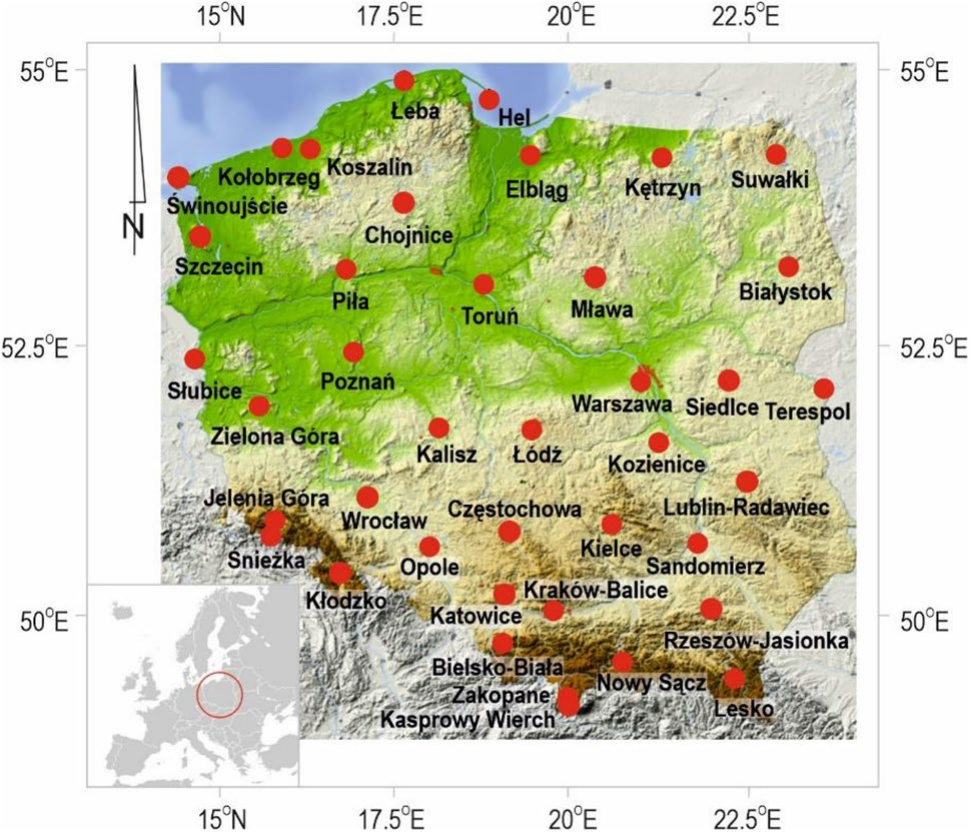
The distribution of the selected 40 synoptic stations in Poland, (based on data: https://egms.land.copernicus.eu/, modified in SURFER 25.2.259).

The present research utilizes daily meteorological data for the period 1966–2020 gathered from 40 synoptic stations located in Poland (Fig. 1). Synoptic stations of best quality are those operating according to WMO standards where manual observations were performed by qualified specialists, stations were not relocated, and data were complete with only single gaps permissible in the whole studied period of 1966–2020. Daily values were derived from synoptic data collected every three hours that comprise manual observations of weather phenomena (every 3 or 1 h), precipitation amount (every 6 or 12 h), air temperature, station level pressure and wind speed (every 3 h). Subsequently, the sub-daily data were used to calculate the daily values of the above mentioned parameters and additionally the mean wet bulb temperature, water vapor pressure, relative humidity, dew point temperature, minimum and maximum temperatures. The precipitation phase was identified based on the notation of weather phenomena coded according to standards in WMO Manual on codes (2019). Detailed information on the procedure of precipitation phase identification (solid, liquid, mixed) based on coded weather can be found in Łupikasza and Małarzewski (2023).

The quality of meteorological data used in this study underwent checking with Standard Normalised Homogenity Test (relative test) applied to daily and monthly values. Out of 60 synoptic stations we selected 40 stations with good quality data which were further checked with respect to outliers and extremes. Extreme precipitation totals were all verified and proved to be correct based on synoptic maps and known flood events. The data on precipitation phase from visual observations are less prone to inhomogeneities due to station relocations, however, a monthly number of days with precipitation phases were correlated between neighbouring stations. No evident inhomogeneities were detected. Data on the daily precipitation phase were utilized in the construction of classification models. In this study, the data used for classifying precipitation into rainfall and snowfall spans from November to April of the following year, as snowfall mainly occurs during this period, and the remaining months are not included in the analysis. In addition, the mixed precipitation was excluded from our analysis as its involvement exhibited poor classification performance in preliminary results. Trace precipitation was excluded from analysis due to its low environmental significance and higher susceptibility to erroneous identification of precipitation type^45^. At each station, the data was split into a training set (70%) and a test set (30%).

### Random Forest

Random Forest (RF) (Breiman, 2001) is a machine-learning model increasingly used for classification. To apply the RF model for classifying precipitation phases, several procedural steps must be taken. Firstly, a dataset consisting of meteorological variables and precipitation phases needs to be collected. The meteorological variables included mean wet bulb temperature, water vapor pressure, relative humidity, dew point temperature, mean air temperature, minimum air temperature, maximum air temperature, precipitation amount, station level pressure, and wind speed**,** while the precipitation phases include no precipitation, rainfall, snowfall, and mixed phase. However, this study focused solely on the classification of rainfall and snowfall, thus only samples corresponding to rainfall and snowfall were retained. This dataset is then split into training (70%) and testing (30%) sets.

Next, the RF model is trained using the training set. This involves constructing multiple decision trees based on the meteorological variables and precipitation phases in the training set. Each decision tree splits the data based on a particular meteorological variable, and the final prediction is made based on the majority vote of the individual decision trees. After the model is trained, it is tested using the testing set. After making predictions on the testing set, the model's accuracy is determined by comparing its predicted precipitation phases with the actual precipitation phases. The evaluation metric for model performance, referred to as 'accuracy', is detailed in Section “Evaluation metric”.

In summary, the RF model can be used to classify precipitation types by training the model on a dataset of meteorological variables and precipitation phases, constructing decision trees based on the variables, and making predictions based on the majority vote of the decision trees.

In this study, the optimal values of *mtry* (number of variables randomly sampled at each split) and *ntree* (number of trees) for each selected meteorological station were determined using the grid search method. These parameters are hyperparameters in the RF model, which are used to control the behavior of the algorithm. Specifically, the value of *mtry* ranged from 1 to 10 with a step of 1, and the value of *ntree* ranged from 1 to 4000 with a step of 100. The default settings were used for other model parameters. In this study we chose RF due to its stability with reference to multicollinearity^53^.

### Optimized threshold temperature approach

The daily mean threshold temperature approach is a method of classifying precipitation as either rainfall or snowfall based on the air temperature at which the precipitation falls. If the daily mean air temperature exceeds the local threshold temperature, then the amount of daily precipitation is categorized as liquid, and conversely, if the daily average air temperature is below or equal the threshold, the precipitation is classified as solid. This approach, which is also known as the scoring method, is commonly used in meteorology and weather forecasting to predict the precipitation type^56,57^.

In this study, we developed an R programming code to search for the optimal mean threshold temperature (*optTa*) for each of the 40 meteorological stations across Poland. This programming code automatically identifies the *optTa* for each station by analyzing the historical data on precipitation phase. The *optTa* was determined using the scoring method^58^ that identifies the lowest error of misclassified precipitation types. Specifically, the code searches for the best value of *Ta* (mean air temperature threshold) within the range of − 2.0 to 2.0 °C, with an increment of 0.1 °C. The *optTa* was selected based on the highest accuracy of the classification. We used an identical method to determine the optimal mean wet-bulb threshold temperature (*optTw*), however the range − 3.0 to 2.0 °C was established for *Tw* (mean wet-bulb temperature) because wet-bulb temperature is by definition lower or equal to air temperature. Wet-bulb temperature is closer to the surface air temperature of a falling hydrometeor than *Ta*^59^, therefore is considered to be a better parameter for discriminating precipitation phases^41,42,56^.

In this study, the aforementioned method has been formulated to compute *optTa* and *optTw* for individual weather stations. This approach is more advantageous compared to amalgamating stations into larger regions or entire countries, as previously seen in various reports.

### Evaluation metric

In this study, we used the below metric to evaluate the accuracy of the classification model:

$${\text{Accuracy}} = \left( {{\text{TP}} + {\text{TN}}} \right)/\left( {{\text{TP}} + {\text{FP}} + {\text{TN}} + {\text{FN}}} \right),$$

where TP—True Positive, the model correctly predicted that the instance belongs to the positive class (rainfall or snowfall), TN—True Negative, the model correctly predicted that the instance belongs to the negative class, FP—False Positive, the model incorrectly predicted that the instance belongs to the positive class, FN—False Negative, the model incorrectly predicted that the instance belongs to the negative class.

## Results and discussion

### Optimizing threshold temperatures using *Ta* and *Tw*

**Figure 2:**
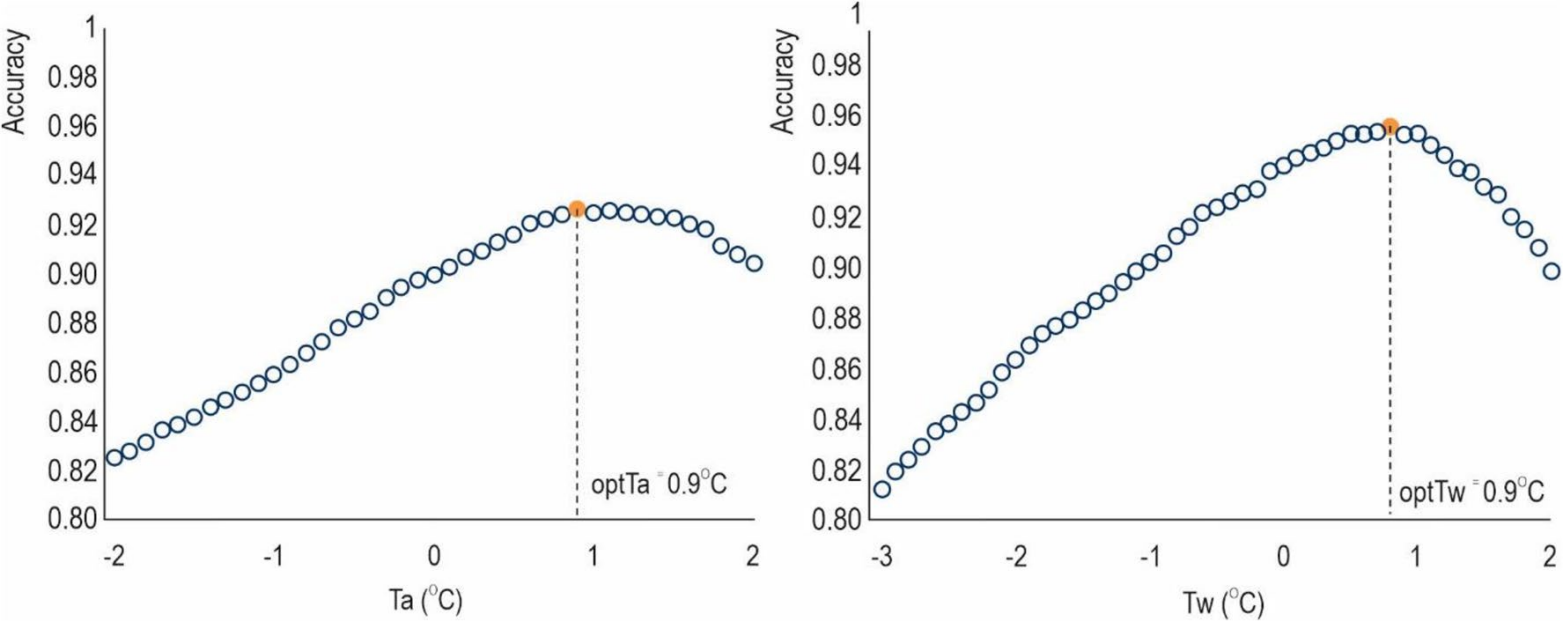
An illustration of the process for searching threshold temperatures using *Ta* (the left figure) and *Tw* (the right figure) is demonstrated by selecting the Kołobrzeg meteorological station. All 40 meteorological stations were processed similarly to find the *optTa* and *optTw*.

To determine the optimal threshold mean temperature (*optTa*), a search was conducted within the range of − 2.0 to 2.0 °C for each meteorological station. The search process for *optTa* values at Kołobrzeg station (WMO 12100) is pictured in Fig. 2. During the search, mean air temperature values between − 2.0 and 2.0 °C were examined sequentially, with an increment of 0.1 °C and, the accuracy of snowfall and rainfall classification (as defined in Section “Evaluation metric”) was calculated at every term and compared. The *optTa* value was selected as the *Ta* that yielded the highest accuracy for snowfall and rainfall classification. This procedure was repeated separately for each of the 40 synoptic stations, thus 40 corresponding *optTa* values were identified and are presented in Table 1.

**Table 1:** The *optTa* and *optTw* values for the 40 meteorological stations located throughout Poland.

WMO no	Station name	Latitude	Longitude	Elevation (m)	*optTa* (°C)	*optTw* (°C)
12 100	Kołobrzeg	54.18	15.58	3	0.9	0.8
12 105	Koszalin	54.20	16.15	33	1.2	0.5
12 120	Łeba	54.75	17.53	2	0.8	0.5
12 135	Hel	54.60	18.82	1	1.3	0.8
12 160	Elbląg	54.17	19.43	40	1.0	0.6
12 185	Kętrzyn	54.07	21.37	108	0.8	0.6
12 195	Suwałki	54.13	22.95	184	0.5	0.3
12 200	Świnoujscie	53.92	14.23	6	0.8	0.7
12 205	Szczecin-Dąbie	53.40	14.62	1	0.9	0.6
12 230	Piła	53.13	16.75	72	0.6	0.5
12 235	Chojnice	53.72	17.55	164	0.7	0.1
12 250	Toruń	53.03	18.58	69	1.0	0.5
12 270	Mława	53.10	20.35	147	0.6	0.4
12 295	Białystok	53.10	23.17	148	0.9	0.6
12 310	Słubice	52.35	14.60	21	0.7	− 0.1
12 330	Poznań-Ławica	52.42	16.85	83	1.0	0.3
12 375	Warszawa-Okęcie	52.17	20.97	106	0.9	0.5
12 385	Siedlce	52.18	22.25	152	0.6	0.4
12 399	Terespol	52.07	23.62	133	0.9	0.4
12 400	Zielona Góra	51.93	15.53	192	1.1	0.4
12 424	Wrocław-Strachowice	51.10	16.88	120	1.2	0.4
12 435	Kalisz	51.78	18.08	138	0.6	0.0
12 465	Łódź-Lublinek	51.73	19.40	187	1.0	0.4
12 488	Kozienice-Nowiny	51.57	21.55	123	0.9	0.4
12 495	Lublin-Radawiec	51.22	22.40	238	0.9	0.4
12 500	Jelenia Góra	50.90	15.80	342	1.2	0.2
12 510	Śnieżka	50.73	15.73	1603	− 0.1	0.1
12 520	Kłodzko	50.43	16.62	356	1.2	0.7
12 530	Opole	50.63	17.97	163	1.1	0.7
12 550	Częstochowa	50.82	19.10	293	0.7	0.3
12 560	Katowice—Muchowiec	50.23	19.03	278	1.2	0.6
12 566	Kraków-Balice	50.08	19.80	237	0.9	0.5
12 570	Kielce-Suków	50.82	20.70	260	0.9	0.4
12 580	Rzeszów-Jasionka	50.10	22.05	206	1.3	0.4
12 585	Sandomierz	50.70	21.72	217	0.8	0.5
12 600	Aleksandrowice	49.80	19.00	398	1.9	0.6
12 625	Zakopane—Równia Krupowa	49.30	19.95	857	1.5	0.5
12 650	Kasprowy Wierch	49.23	19.98	1991	1.9	0.4
12 660	Nowy Sącz	49.62	20.70	306	1.5	0.5
12 690	Przemyśl	49.47	22.33	279	1.5	0.6

Excluding the Śnieżka station, the *optTa* values ranged between 0.5 and 1.9 °C, and it reached (a minimum of) − 0.1 °C at the Śnieżka station, and (a maximum of) 1.9 °C, at both the Kasprowy Wierch and Aleksandrowice stations. According to Jennings et al. (2018), 95% of stations in the Northern Hemisphere had a threshold temperature ranging from − 0.4 to 2.4 °C. Regional or continental scale studies indicated the threshold temperature between − 1.0 and 2.5 °C^39,40,60^ thus the assessed *optTa* values for Poland fall into the hemisphere ranges. The *optTw* values varied between − 0.1 °C at the Słubice station and 0.8 °C at Kołobrzeg and Hel stations. The *Tw* thresholds were lower than *Ta*, as the wet-bulb temperature, by definition, is lower than the air temperature. Moreover, *optTw* range was narrower than *optTa*, indicating better performance than *Ta*, which was also found in^41,42,56^. The only exception is Śnieżka mountain station considered the station with most harsh climate conditions where both threshold temperatures are very low and *optTa* < *optTw*, although for single days wet-bulb was checked to be lower than air temperature. Noting that at this station, the classification accuracy at *optTw* = − 0.1 °C is 0.9551, which is only slightly different from the accuracy of 0.9562 at *optTw* = 0.1 °C.

Due to their close dependence on local atmospheric and geographical conditions, the *optTa* and *optTw* thresholds for distinguishing precipitation types demonstrated spatiotemporal variations^61^. Hynčica and Huth (2019) reported a significant variation in *Ta* values across northern Europe and even within a small area like the Czech Republic. The use of spatially uniform *Ta* values in land surface models may produce imprecise estimations of precipitation phases^61^, particularly in areas with variable topography. The temperature threshold for distinguishing between snowfall and rainfall over high-elevation regions has been observed to increase. In Poland, the *optTa* value for Kasprowy Wierch station located at 1991 m, is 1.9, while for Zakopane (857 m), Aleksandrowice (398 m), Kłodzko (356 m), and Jelenia Góra (342 m) are 1.9 °C, 1.5 °C, 1.2 °C, and 1.2 °C, respectively which indicated a 0.7 °C range of variability. This tendency can be explained by the lower air pressure at the higher elevations and thinner air, resulting in less resistance on the snow crystals. As a result, the crystals reach the ground faster, and absorb less heat from the ambient atmospheric environment, thereby allowing them to maintain their original state as snow at higher air temperatures at higher elevations^41^. However, there may be exceptions to this pattern, as precipitation type is also influenced by various other factors. The accurate *optTa* and *optTw* values for Polish synoptic stations are tabulated in Table 1 and can be used to discriminate between the liquid and solid phases. The range of variability in optimal air temperatures reached 1.9 °C for *optTa* and 0.8 °C for *optTw*. Such a wide range of variability indicates that a single threshold temperature used to discriminate snowfall from rainfall over the entire country can generate inaccurate classification.

### Selection of meteorological variables best for discrimination of precipitation phases

Ten meteorological variables were selected to check their importance for discriminating precipitation phases with the Multiple Input Random Forest (MIRF) classification model.

**Figure 3:**
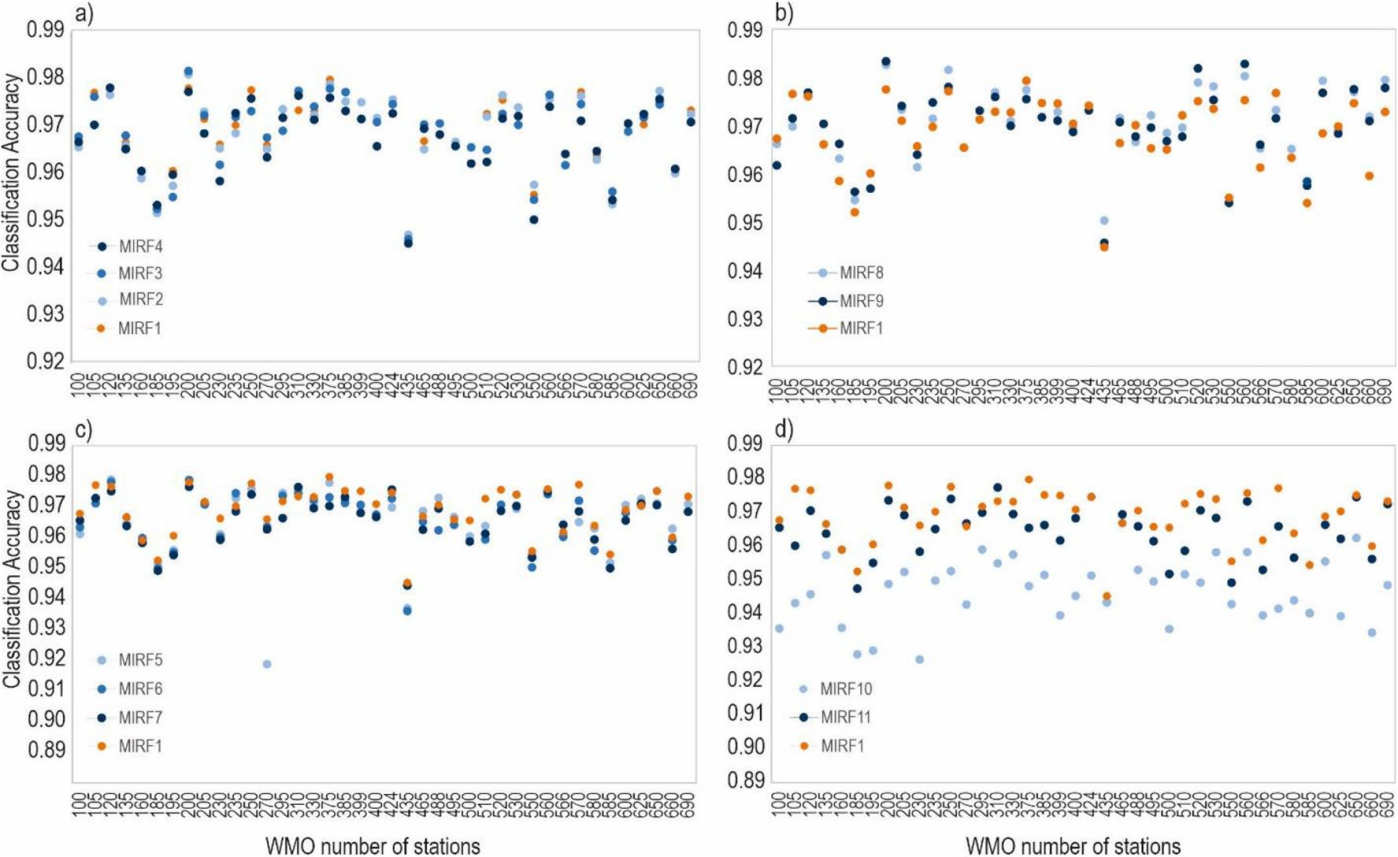
Comparing the accuracy of various MIRF models in identifying the most effective meteorological factor for distinguishing between snowfall and rainfall. (**a**) Comparison performance of models MIRF1, MIRF2, MIRF3, and MIRF4; (**b**) Comparison performance of models MIRF1, MIRF8, and MIRF9; (**c**) Comparison performance of models MIRF1, MIRF5, MIRF6, and MIRF7; (**d**) Comparison performance of models MIRF1, MIRF10, and MIRF11.

Initially, all ten variables were used as input in the MIRF1 model (Table 2). Subsequently, to obtain a simpler and more effective model, the inputs were reduced if the classification performance did not deteriorate. The procedure was repeated as long as all variables that had no impact on modeling quality were eliminated. For example, at the first check wind speed was removed as an input variable to construct the MIRF2 model. The performance of MIRF2 was compared with that of MIRF1, and no significant difference was observed between the two models. Therefore, wind speed was considered not important in this classification model and was removed. A similar process was conducted to remove station level pressure in MIRF3 and precipitation amount in MIRF4. Figure 3a presents the classification performance after removing wind speed, station level pressure, and precipitation amount and shows no significant difference among the four models (MIRF1, MIRF2, MIRF3, and MIRF4).

**Table 2:** MIRF models and their corresponding input variables.

Model	Input	Removed variables
MIRF1	Mean wet bulb temperature, water vapor pressure, relative humidity, dew point temperature, mean air temperature, minimum air temperature, maximum air temperature, precipitation amount, station level pressure, and wind speed	N/A
MIRF2	Mean wet bulb temperature, water vapor pressure, relative humidity, dew point temperature, mean air temperature, minimum air temperature, maximum air temperature, precipitation amount, and station level pressure	Wind speed
MIRF3	Mean wet bulb temperature, water vapor pressure, relative humidity, dew point temperature, mean air temperature, minimum air temperature, maximum air temperature, and precipitation amount	Wind speed, station level pressure
MIRF4	Mean wet bulb temperature, water vapor pressure, relative humidity, dew point temperature, mean air temperature, minimum air temperature, and maximum air temperature	Wind speed, station level pressure, precipitation amount
MIRF5	Mean wet bulb temperature, water vapor pressure, relative humidity, dew point temperature, mean air temperature, and minimum air temperature	Wind speed, station level pressure, precipitation amount, maximum air temperature
MIRF6	Mean wet bulb temperature, water vapor pressure, relative humidity, dew point temperature, mean air temperature, and maximum air temperature	Wind speed, station level pressure, precipitation amount, minimum air temperature
MIRF7	Mean wet bulb temperature, water vapor pressure, relative humidity, dew point temperature, minimum air temperature, and maximum air temperature	Wind speed, station level pressure, precipitation amount, mean air temperature
MIRF8	Water vapor pressure, relative humidity, dew point temperature, mean air temperature, minimum air temperature, and maximum air temperature	Wind speed, station level pressure, precipitation amount, mean wet bulb temperature
MIRF9	Water vapor pressure, relative humidity, mean air temperature, minimum air temperature, and maximum air temperature	Wind speed, station level pressure, precipitation amount, mean wet bulb temperature, dew point temperature
MIRF10	Relative humidity, dew point temperature, mean air temperature, minimum air temperature, and maximum air temperature	Wind speed, station level pressure, precipitation amount, mean wet bulb temperature, water vapor pressure
MIRF11	Water vapor pressure, mean air temperature, minimum air temperature, and maximum air temperature	Wind speed, station level pressure, precipitation amount, dew point temperature, mean wet bulb temperature, relative humidity
The final model (hereafter MIRF)	Water vapor pressure, relative humidity, mean air temperature, minimum air temperature, and maximum air temperature	Wind speed, station level pressure, precipitation amount, dew point temperature, mean wet bulb temperature

Similarly, MIRF5 was built by excluding the maximum air temperature, but the model's performance decreased, particularly at the Mława (WMO#12270) and Kalisz (WMO#12435) stations (Fig. 3c). As a result, the maximum air temperature was retained, indicating that it is an important factor when discriminating snowfall from rainfall at the aforementioned stations. Next, in MIRF6 and MIRF7, the minimum air temperature and mean air temperature were removed, respectively, but the model's performance deteriorated. Therefore, it was decided to keep these variables.

Although several studies^41,61^ emphasized the importance of *Tw* in rainfall/snowfall classification, surprisingly, the MIRF8 performance was found to be better when the wet-bulb temperature (*Tw*) was removed (Fig. 3b). However, our results did not eliminate *Tw* as an efficient predictor of snowfall as indicated in Section “Optimizing threshold temperatures using Ta and Tw”. The negative effect of *Tw* on model performance can be explained by the fact that *Tw* was calculated based on air temperature, relative humidity, air pressure, and saturated vapor pressure, all of which were included in the set of input variables thus leading to the problem of multicollinearity which complicated the MIRF. In the further steps, dew point temperature in MIRF9 was eliminated.

Removing either water vapor pressure (in MIRF10) or relative humidity (in MIRF11) reduced the classification accuracy (Fig. 3d), and thus the two variables played an essential role in the model. Finally, five variables were retained as input variables for the classification model, including water vapor pressure, relative humidity, mean air temperature, maximum air temperature, and minimum air temperature. These results contribute to previous studies that found average air temperature and relative humidity or dew point temperature^40,60,62,63^ as crucial variables for the discrimination of snowfall from rainfall. Many studies suggest a better performance of wet-bulb temperature than other temperature-based methods^41,42,61^, however, it does not apply to the MIRF method.

The model that utilizes those 5 variables was referred to as MIRF and its performance was compared with the *optTa* and *optTw* methods (in Sub-section “The mean threshold temperature methods versus Single Input Random Forest Model (SIRF)”).

### Comparison of threshold temperature methods and the Random Forest (RF)

#### The mean threshold temperature methods versus Single Input Random Forest Model (SIRF)

**Figure 4:**
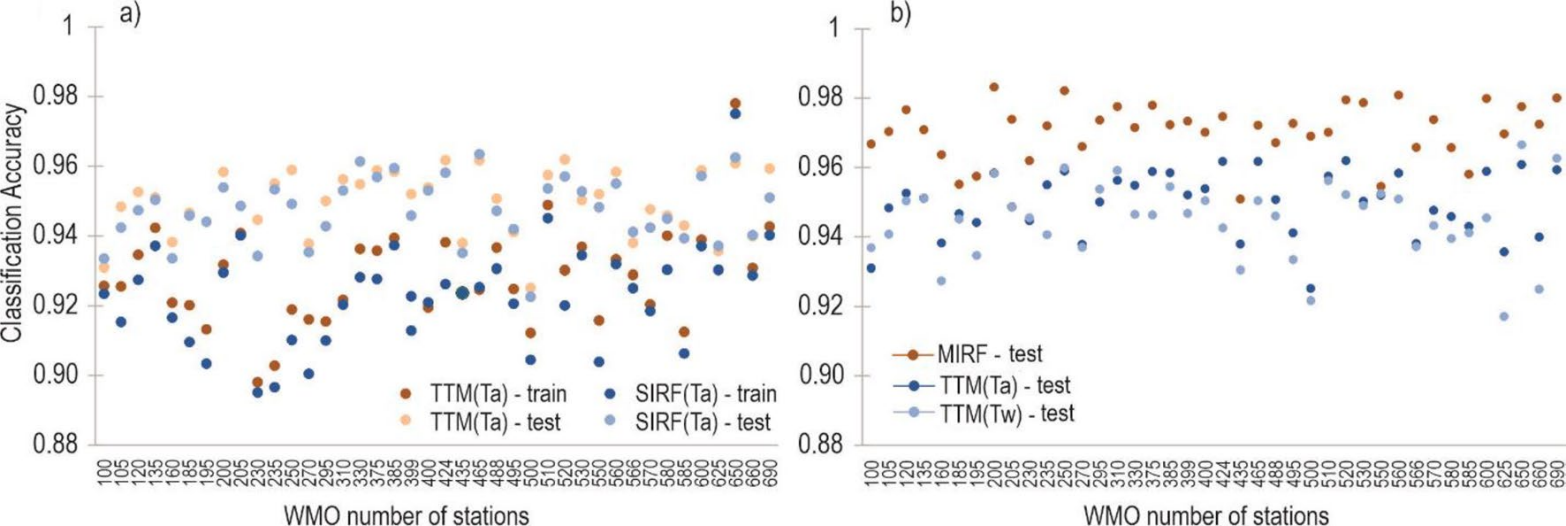
Threshold temperature methods versus Single Input Random Forest Model (SIRF), and Multi Input Random Forest (MIRF); (**a**) Classification accuracy of TTM(*Ta*) and SIRF(*Ta*) in the train and test datasets; (**b**) Classification accuracy of TTM(*Ta*), TTM(*Tw*), and MIRF in the test dataset.

The performance of the mean threshold temperature method and the Single Input Random Forest (SIRF) method, which utilized only the mean air temperature as an input for the RF model were compared to assess the capability of the two methods when using the same input parameter. Figure 4a illustrates that the performance of the SIRF model was slightly worse than the mean threshold temperature method in both training and testing. This suggests that the benefits of machine learning methods such as RF are only effective and can be leveraged to their full potential when used for modelling complicated and non-obvious relationships between a set of variables. This is evidenced in Fig. 4b which shows that MIRF outperformed the threshold temperature methods, both temperature threshold method-based average temperature-TTM(*Ta*) and temperature threshold method-based wet bulb temperature-TTM(*Tw*).

The process of precipitation formation is influenced by many meteorological factors, so it is reasonable that MIRF, which involves more variables, achieved better results in rainfall/snowfall classification. The issue is that MIRF needs to be properly constructed to find reasonable hyper-parameters and to avoid overfitting when used for prediction. At this point, the ability to handle the complicated relationship between rainfall/snowfall and predictors of the RF model can be fully harnessed. In the case of few meteorological variables available, the threshold temperature method is a better choice.

Additionally, it is evident that the threshold temperature method based on *Ta* (TTM(*Ta*)) demonstrated slightly superior performance compared to the temperature threshold method based on *Tw* (TTM(*Tw*)). Specifically, TTM(*Ta*) outperformed TTM(*Tw*) in 29 (72.5%) out of 40 meteorological stations. Overall, the classification accuracy of these two methods did not differ significantly.

### Assessing the accuracy of the monthly-scale rainfall/snowfall classification model

**Figure 5:**
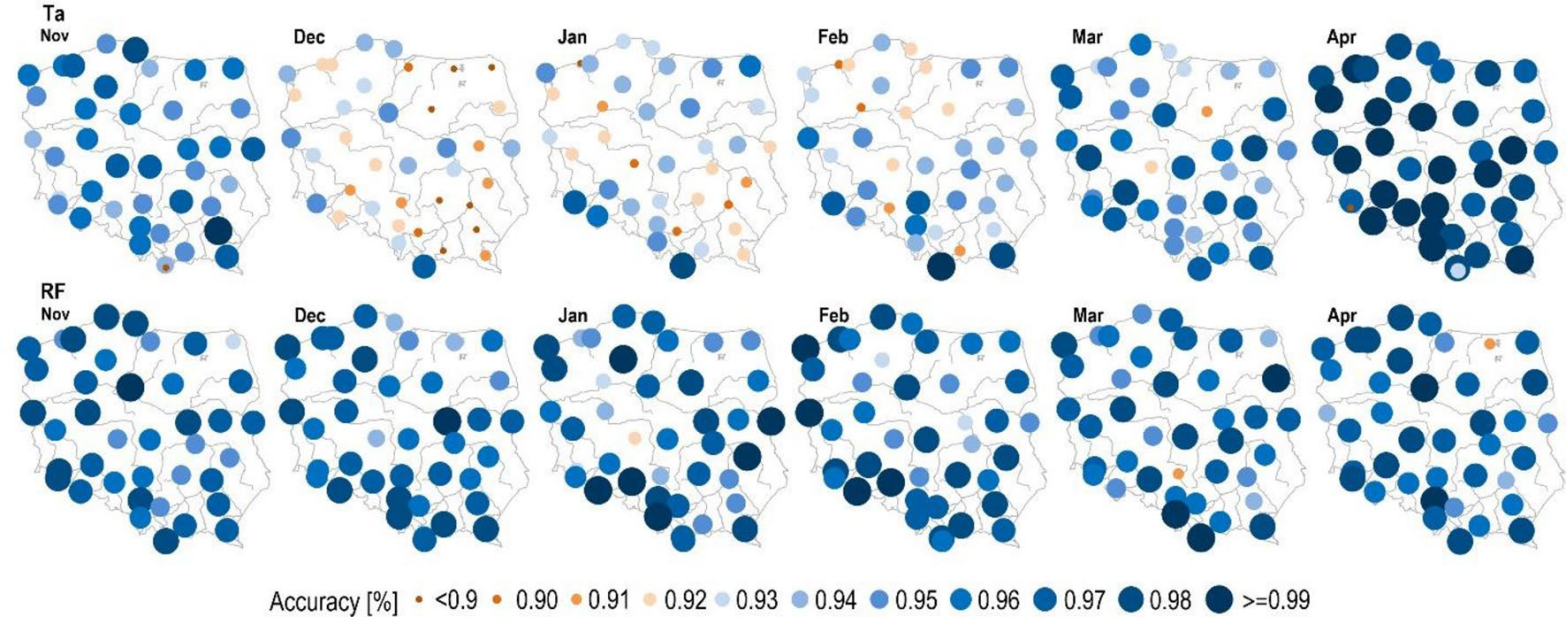
The spatial distribution of the accuracy of the TTM(*Ta*) (first row) and MIRF method (second row) for rainfall /snowfall classification, (software: SURFER 25.2.259).

The accuracy of the analyzed methods for discriminating snowfall and rainfall exhibited seasonal variations (Fig. 5). Multiple Input Random Forest (MIRF) outperforms the Threshold Temperature Method (TTM(*Ta*)) in terms of classification accuracy in December, January, February, and March. In November, TTM(*Ta*) and MIRF exhibited comparable classification performance, with TTM(*Ta*) even surpassing RF in April. The RF outperformed TTM(*Ta*) between December and March, i.e. in the months with low air temperature when the occurrence of snowfall strongly depends also on the set of other variables that, as recognized in Section “Selection of meteorological variables best for discrimination of precipitation phases”, include water vapor pressure, relative humidity, maximum air temperature, and minimum air temperature. In November, and April air temperature is relatively high in Poland thus probably having an enhanced influence on solid precipitation occurrence. Another possibility is that the atmosphere is more unstable in the shoulder seasons leading to more cumuliform (unstable) rather than stratiform (stable) conditions during precipitation events (this would change lapse rates which could affect the energy transfer for melting and refreezing while hydrometers fall from the cloud to ground at a given surface air temperature). In fact, these relationships are still not fully understood. Moreover, in April the number of snowfall events was much lower than the number of rainfall events. In this month, TTM(*Ta*) could be more efficient than MIRF because machine learning algorithms such as Random Forest require a large number of samples to be trained effectively and learn from past data. The above findings provide insights into selecting the appropriate model for each month with snowfall.

**Figure 6:**
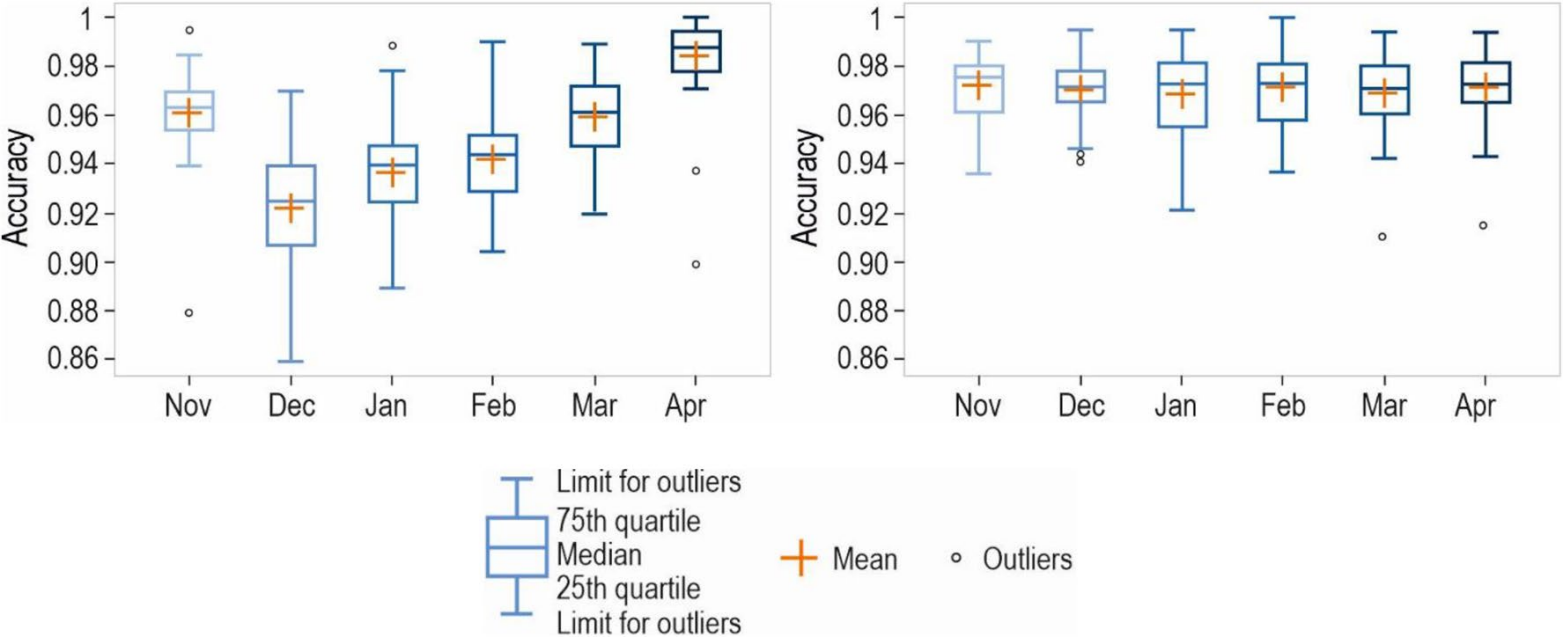
Displays boxplots that depict the accuracy of rainfall/snowfall classification at 40 stations across Poland, presented by month. The boxplot on the left shows the results of the TTM(*Ta*), whereas the boxplot on the right shows the results of MIRF. The orange plus sign indicates the mean value.

Overall, RF demonstrated consistent classification performance across all months, with an average classification accuracy ranging only from 0.97 to 0.97. In contrast, the classification performance of TTM(*Ta*) exhibited wider variation, ranging from 0.92 to 0.98. On average, the classification performance of TTM(*Ta*) increases in the following order: December, January, February, March, November, and reaches its highest level in April (Fig. 6). It should be noted that the above results were extracted from only one model for all the data, rather than building a separate model for each month of the year. Constructing separate models for each month of the year may yield higher results but could be challenging for months with a low number of snowfalls, such as April and November. In this context, choosing MIRF remains a good option. The main text of the research paper only presents information about accuracy. Precision, recall, and F1_score can be found in the Supplementary.

## Conclusion and outlook

Although precipitation phase is important for studying climate change, climate modelling and weather forecasting, discrimination between snowfall and rainfall is still a problem due to lack of observation, length of chronological series and reducing visual observations. The RF model developed in this study to classify the precipitation phases is based on daily values of selected meteorological variables measured at the station level and validated based on high-quality data from visual observations performed at 40 synoptic stations in Poland in the period 1966–2020. Additionally, we employed two other commonly used methods: the threshold mean temperature method and the threshold wet-bulb temperature method. Each of these methods relies solely on a single meteorological variable.

The results of the MIRF model are very promising and performed the best among the three methods. The accuracy of RF snowfall classification ranged from 91 to 100%. The mean threshold temperature and wet–bulb threshold temperatures showed similar classification performance.

Several experiments were also conducted to select the best predictors for the RF classification. The model revealed water vapor pressure as a crucial meteorological parameter for discriminating precipitation phase because it combines information on air temperature. Subsequently, the following variables also contributed to increasing the accuracy of the classification model: relative humidity, mean air temperature, minimum air temperature, and maximum air temperature. Surprisingly, wet–bulb temperature did not contribute to improving the performance of the MIRF classification model due to multicollinearity related to the method of *Tw* calculation. Furthermore, the MIRF model could be simplified without impacting accuracy by eliminating wind speed, station level pressure, precipitation amount, and dew point temperature. The findings are useful for future studies in selecting an appropriate model and predictors for classifying rainfall and snowfall.

A classification model based on RF has been developed and can be utilized to identify precipitation phases in meteorological stations located in Poland where information on precipitation phase is not available or visual observations are exchanged with automatic measurements. This model can be further extended to other European countries to detect precipitation phases.

Under climate change conditions, the processes triggering the formation of precipitation phases become more complex. Therefore, continuous observation and improvement of the classification models for precipitation types are necessary to enhance the effectiveness of the models in prediction.

In this study, the mixed precipitation phase exhibited poor classification performance in preliminary results. Therefore, it was excluded from our analysis, as no added value could be contributed to its classification. To improve the performance of the classification model and address the imbalance phenomena observed at some stations, techniques such as oversampling or undersampling can be employed in future research. However, due to the scope of this study, we did not implement these techniques. The poor predictive performance of sleet is attributed to its infrequent occurrence^44^ and the complexity of its formation process. The classification of mixed solid–liquid precipitation is known to be challenging and results in low forecast performance, as reported in several studies^34,53,64^.

### Supplementary Information

Supplementary Information.

## Data Availability

The data that support the findings of this study are available from the corresponding author, upon reasonable request.
