# Supplementary material for: Classification of precipitation types in Poland using machine learning and threshold temperature methods
Source: Sci Rep. 2023 Nov 25;13:20750. doi: 10.1038/s41598-023-48108-2 (PMC10676369; doi:10.1038/s41598-023-48108-2)
Supplement: Supplementary file 1 — Supplementary Information. [file 41598_2023_48108_MOESM1_ESM.docx]

**C**l**assification of Precipitation Types in Poland Using Machine Learning and Threshold Temperature Methods**

Quoc Bao Pham^1^, Ewa Łupikasza^1^*, Małarzewski Łukasz^1^

^1^Faculty of Natural Sciences, Institute of Earth Sciences, University of Silesia in Katowice, Będzińska street 60, 41-200, Sosnowiec, Poland

*Correspondence: ewa.lupikasza@us.edu.pl

Supplementary

Table A. Precision, Recall, and F1_score at 40 meteorological stations in the test period.

| **Station_ID** | **Precision** | **Recall** | **F1_Score** |
| --- | --- | --- | --- |
| 100 | 0.9795 | 0.9820 | 0.9807 |
| 105 | 0.9748 | 0.9867 | 0.9807 |
| 120 | 0.9852 | 0.9842 | 0.9847 |
| 135 | 0.9704 | 0.9903 | 0.9802 |
| 160 | 0.9582 | 0.9853 | 0.9715 |
| 185 | 0.9645 | 0.9704 | 0.9674 |
| 195 | 0.9556 | 0.9713 | 0.9634 |
| 200 | 0.9883 | 0.9910 | 0.9896 |
| 205 | 0.9796 | 0.9888 | 0.9842 |
| 230 | 0.9644 | 0.9875 | 0.9758 |
| 235 | 0.9834 | 0.9775 | 0.9804 |
| 250 | 0.9809 | 0.9952 | 0.9880 |
| 270 | 0.9746 | 0.9733 | 0.9739 |
| 295 | 0.9710 | 0.9867 | 0.9788 |
| 310 | 0.9782 | 0.9951 | 0.9866 |
| 330 | 0.9774 | 0.9856 | 0.9815 |
| 375 | 0.9800 | 0.9871 | 0.9835 |
| 385 | 0.9710 | 0.9882 | 0.9795 |
| 399 | 0.9775 | 0.9803 | 0.9789 |
| 400 | 0.9735 | 0.9860 | 0.9797 |
| 424 | 0.9745 | 0.9922 | 0.9833 |
| 435 | 0.9721 | 0.9612 | 0.9666 |
| 465 | 0.9685 | 0.9910 | 0.9796 |
| 488 | 0.9627 | 0.9917 | 0.9770 |
| 495 | 0.9662 | 0.9885 | 0.9772 |
| 500 | 0.9615 | 0.9904 | 0.9757 |
| 510 | 0.8641 | 0.9036 | 0.8834 |
| 520 | 0.9718 | 0.9952 | 0.9834 |
| 530 | 0.9783 | 0.9922 | 0.9852 |
| 550 | 0.9466 | 0.9853 | 0.9656 |
| 560 | 0.9789 | 0.9907 | 0.9847 |
| 566 | 0.9667 | 0.9800 | 0.9733 |
| 570 | 0.9717 | 0.9877 | 0.9796 |
| 580 | 0.9669 | 0.9804 | 0.9736 |
| 585 | 0.9583 | 0.9780 | 0.9680 |
| 600 | 0.9745 | 0.9914 | 0.9829 |
| 625 | 0.9470 | 0.9708 | 0.9588 |
| 650 | 0.7227 | 0.9348 | 0.8152 |
| 660 | 0.9637 | 0.9922 | 0.9778 |
| 690 | 0.9722 | 0.9906 | 0.9813 |
